# Four New Cases of SLC35A2-CDG With Novel Mutations and Clinical Features

**DOI:** 10.3389/fgene.2021.658786

**Published:** 2021-05-27

**Authors:** Kuerbanjiang Abuduxikuer, Jian-She Wang

**Affiliations:** Department of Hepatology, Children’s Hospital of Fudan University, Shanghai, China

**Keywords:** SLC35A2-CDG, UDP-galactose transporter, CDG-IIm, congenital disorders of glycosylation, Chinese

## Abstract

SLC35A2-CDG is a rare type of X-linked CDG with more than 60 reported cases. We retrospectively analyzed clinical phenotypes and *SLC35A2* genotypes of four cases of SLC35A2-CDG from four unrelated families of Han ethnicity in China. All patients had infantile onset epilepsies that were completely or partly resistant to multiple anti-epileptic medications or ketogenic diet. Three patients had severe developmental delay. All patients were female patients carrying *de novo* deleterious mutations in *SLC35A2* (NM_001042498.2) gene, including one canonical splice-site mutation (c.426+1G > A), one large deletion (c.-322_c.274+1del), and two frameshift mutations leading to premature stop codon (c.781delC/p.Arg289ValfsTer88 and c.601delG/p.Ala201GlnfsTer148). Novel clinical features in some of our patients include anemia, hypertriglyceridemia, hypertonia, small ears, extra folds on earlobes, and maternal oligohydramnios or hypothyroidism during pregnancy. In one patient, concomitant Marfan syndrome was confirmed for having positive family history, carrying a heterozygous known disease-causing mutation in FBN1 gene (c.7240C > T/p.Arg2414Ter), and presence of typical features (rachnodactyly, ventrical septal defect, and mitral valve regurgitation). In conclusion, we expanded clinical phenotype and genetic mutation spectrum of SLC35A2-CDG by reporting four new cases with novel pathogenic variants and novel clinical features.

## Introduction

Congenital disorder of glycosylation (CDG) is a rapidly expanding group of metabolic disorders of glycan biosynthesis or assembly. Most of more than 140 types of CDGs discovered so far have neurologic phenotype, and can affect any other organs or systems depending on the specific CDG types (Chang et al., 2018; Ng and Freeze, 2018). Located in Xp11.23, *SLC35A2* gene (OMIM^∗^314375) encodes solute carrier family 35 member 2 protein (UDP−galactose transporter, SLC35A2) in humans responsible for transporting UDP-galactose from the cytosol to lumens of Golgi apparatus and endoplasmic reticulum for glycosylation (Ishida et al., 1996; Miura et al., 1996). Inactivation of SLC35A2 gene in mammalian cell models caused total absence of UDP−galactose traffic into the Golgi apparatus leading to synthesis of truncated glycans lacking galactose (Brändli et al., 1988; Ishida et al., 1999). In humans, SLC35A2-CDG (OMIM#300896, formerly known as CDG type IIm) is caused by heterozygous *de novo* mutations in *SLC35A* gene and mostly affect female gender due to X−chromosome inactivation. Clinical phenotypes include infantile-onset seizure, global development delay, facial dysmorphism, abnormal liver function, and skeletal abnormalities. Since the first report of SLC35A2-CDG in 2013 (Ng et al., 2013), 54 *de novo* variants from 63 cases have been reported in the literature (Kodera et al., 2013; Ng et al., 2013, 2019; EuroEPINOMICS-Res Consortium, Epilepsy Phenome/Genome Project, and Epi4K Consortium., 2014; Dörre et al., 2015; Bosch et al., 2016; Lopes et al., 2016; The Undiagnosed Disease Network, 2016; Evers et al., 2017; Kimizu et al., 2017; Westenfield et al., 2018; Yates et al., 2018; Miyamoto et al., 2019; Vals et al., 2019). However, no patient has been reported from mainland China in English literature. Here we report four new patients with typical features of SLC35A2-CDG from China, and expand genotypic as well as phenotypic features with novel findings.

## Materials and Methods

### Patient Recruitment and Literature Analysis

The Ethics Committee of the Children’s Hospital of Fudan University approved this study. We retrospectively analyzed clinical phenotype and *SLC35A2* genotype of four cases (P1–P4) from four unrelated families. Three patients (P1, P2, and P3), were treated in the Department of Hepatology, Children’s Hospital of Fudan University from January, 2017 to July, 2020, and were asked to fill out questionnaire with phenotypes related to SLC35A2-CDG. P2 and P3 signed an informed consent before recommending oral D-galactose therapy. Pubmed searches were conducted at 11 June 2020 using key words such as SLC35A2, UDP-galactose transporter (species were filtered for humans), but no reports from Mainland China were found. We also conducted Chinese language literature search by using the CNKI (China Knowledge Resource Integrated Database^1^) and Wanfang Database^2^ and collected a case report of SLC35A2-CDG (P4) in Chinese medical literature. We also analyzed all reported cases of SLC35A2-CDG in English literature.

### Sequencing and Bioinformatics Analysis

**FIGURE 1:**
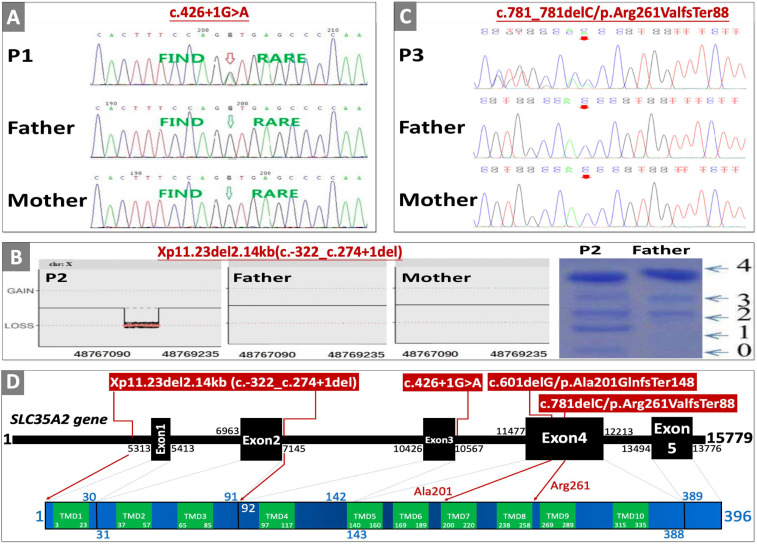
Genetic testing, transferrin isoelectric focusing, cDNA changes, and effects on protein domain. **(A)** Sanger sequencing confirmation of P1. **(B)** Whole exome sequencing copy number variant (CNV) analysis result, and serum transferrin isoelectric focusing results of P2 (0, 1, 2, 3, and 4 are the numbers of sialic acid glycosylated with transferrin corresponding to transferrin not glycosylated with sialic acid, transferrin glycosylated with monosialic acid, disialic acid, trisialic acid, and tetrasialic acid). **(C)** Sanger sequencing confirmation of P3. **(D)** Location of variants on SLC35A2 gene with corresponding amino-acid location and transmembrane domains (TMD); *SLC35A2* gene NCBI reference sequence of NG_034300.1, transcript reference of NM_001042498.2, and protein reference of NP_001035963.1, and GRCh37/hg19 were used for cDNA and amino acid changes. Sanger sequencing confirmation of P4 and parents were published in the Chinese literature (Tian et al., 2020).

SLC35A2 gene variants were identified either through whole exome sequencing (WES), panel sequencing, or CNV-seq analysis by using the NM_001042498.2 transcript and checked against the Human Gene Mutation Database (HGMD^®^) Professional^3^. P1 had trio-WES by FindRare (a commercial genetic testing company^4^, using Illumina platform and NextGene V2.3.4 software) (Figure 1A). P2 had trio-WES with CNV-seq analysis in a commercial genetic testing company (Berry Genomics^5^, using Verita Trekker^®^ mutation site detection system and Enliven^®^ Data Annotation and Interpretation System) (Figure 1B). P3 had Epilepsy panel sequencing with parental confirmation by a commercial medical testing company called Kangso Medical Inspection^6^ (Figure 1C). P4 was reported to be identified through simultaneous WES of the index patient, parents, maternal grandmother, and maternal uncle using Illumina Nextseq 500 sequencer. Novelty of *SLC35A2* gene variants were checked against dbSNP152^7^, 1000 Genome Database^8^, Exome Variant Server^9^, and gnomAD^10^. Location of large deletion on the *SLC35A2* gene is confirmed with Mutalyzer 2.0.30 Position Converter^11^. Pathogenicity of genetic variants were predicted by online tools such as MutationTaster^12^, CADD^13^, SIFT Indel^14^, and M-CAP^15^. Effects of variants on gene splicing were predicted with BDGP/NNSplice^16^, ESE Finder 3.0^17^, Human Splicing Finder 3.1^18^, MutationTaster (see text footnote 12), and ASSP (Alternative splice site predictor^19^). All variants were Sanger confirmed.

## Results

### Case Profiles

All cases were Han Chinese, and the only child born to healthy non-consanguineous parents.

P1 was a full-term female born after uneventful pregnancy (except for mild oligohydramnios), and delivery was uncomplicated except for a lower birthweight (2,350 g, 1st centile by WHO standards). She presented with seizure at the age of 3 months, hypsarrhythmia in electroencephalogram (EEG) indicated infantile spasms. Seizures significantly improved after treatment with topiramate and vigabatrin. However, epilepsy was not controlled despite therapies with multiple anti-epileptic drugs (AEDs), adrenocorticotropic hormone (ACTH), and ketogenic diet. She was diagnosed with infantile spasm, epileptic encephalopathy, and psychomotor developmental delay. Brain MRI at the age of 5 months showed delayed myelination, slight enlargement of bilateral lateral ventricles and frontal subarachnoid spaces. At the age of 8.5 months, she still could not hold her head or roll over, eyes rarely follow objects, and rarely smiles. Weight is on the 8th centile (7.5 kg), while length is less than the 1st centile (59 cm). Other abnormal findings include mild microcytic normochromic anemia (hemoglobin 100 g/L, normal range 110–130 g/L) at the age of 7 months, and hypertriglyceridemia (2.98 mmol/L, normal range 0–1.7 mmol/L) with slight elevation of serum uric acid (412 μmol/L, normal range 140–390 umol/L) at the age of 1 year. Trio-WES identified a *de novo* canonical splice-site variant (c.426+1G > A) in *SCL35A2* gene (NM_001042498.2) (Figure 1A), and chromosome microarray analyses were negative for copy number variants. Normal findings include serum lactose, ammonia, creatine kinase, glucose, pyruvate, transaminases, profiles of amino-acid with acylcarnitine, cholesterol, folate, and vitamin B12 levels. This patient lost to follow-up before taking blood samples for glycosylation studies and recommending oral D-galactose therapy.

P2 is the first child of non-consanguineous parents born after full-term pregnancy (38 weeks and 2 days) with normal birthweight (2,950 g, 26th centile) and length (49 cm, 47th centile). Maternal history was positive for pre-conceptional hyperthyroidism and hypothyroidism during pregnancy. Newborn period was uneventful except for significant hyperbilirubinemia and phototherapy. She presented with hypertonia, developmental delay, and seizures at the age of 5 months. EEG showed hypsarrhythmia and multifocal discharges on both temporal and occipital lobes. Physical examination was positive for increased muscle tone on extremities, smaller left ear with extra folds on both earlobes. Seizures were not responsive to topiramate and sodium valproate, but could be partly controlled with ACTH therapy. EEG at the age of 7 months was abnormal. Other abnormal findings include mild hypertransaminasemia (ALT/AST 42/52U/L, normal range 0–30 U/L), and mild lactic acidosis (3.1 mmol/L, normal range 0–2 mmol/L). Trio-WES was negative, but chromosome microarray analysis identified a *de novo* large deletion leading to the absence of the exon 1 and exon 2 (Xp11.23del2.14kb, c.-322_c.274+1del) in the *SLC35A2* gene. She and her healthy father (as normal control) were screened for carbohydrate deficient transferrin by iso-electric focusing. When compared to her father’s normal profile, P3 had increased levels of transferrin glycosylated with monosialic and asialic acids (Figure 1B). Normal findings include lymphocyte sub-population, creatine kinase, thyroid function test, electrocardiography, brain MRI, chest X-ray, T-spot TB test, full blood count, serum amino-acid with acylcarnitine profiling, and urine organic acids. She received gradual increase of oral D-galactose therapy (a health supplement produced by Vita-World GmbH, Germany^20^) starting with 0.5 g/kg^∗^day (divided into five doses), and adding 0.5 g/kg^∗^day in 6 weeks with a final dosage of 1.5 g/kg^∗^day from the age of 11 months (as recommended by a clinical trial protocol, ClinicalTrials.gov Identifier: NCT02955264). When followed up at the age of 22 months, she achieved seizure control with minimal EEG abnormality with slight improvement in both motor and language skills.

P3 was a full-term female infant with normal birthweight (3,100 g, 38th centile) born to non-consanguineous parents. Neonatal period was uneventful except for phototherapy due to hyperbilirubinemia. She developed seizures at the age of 10 months, EEG results were suggestive of infantile spasm (intermittent hypsarrhythmia with focal sharp waves on temporal-occipital lobe). Infantile spasm improved with ketogenic diet, but there was no improvement on abnormal EEG pattern after 3 months. She was found to have *de novo* frameshift mutation (c.781delC/p.Arg289ValfsTer88) on trio-WES (Figure 1C). Normal test results included liver function test, serum cholesterol and triglyceride levels, renal function test, serum electrolytes, full blood count, serum amino-acids with acylcarnitine profiling, urine organic acids, and brain MRI. Psychomotor developmental milestones were within normal range. After signing an informed consent for glycosylation study and oral D-galactose treatment, parents send blood samples of the child and parents to our institution. Seizure activities are controlled 6 months after galactose therapy.

P4 is a 10-month-old girl recently reported in the Chinese literature (Tian et al., 2020). She was born full-term to non-consanguineous parents after an uneventful first pregnancy and vaginal delivery. She had normal birthweight (3,200 g, 47th centile), length (49 cm, 47th centile), and head circumference (34 cm, 54th centile). She was diagnosed with infantile spasms and developmental delay at the age of 3 months. At the age of 10 months, she was found to have severe global developmental delay (inability to hold her head or sit, Gesell Developmental Schedules DQ score was 15, normal range: >65), hypotonia, ventrical septal defect on echocardiography, hypsarrhythmia on electroencephalography, and mega cisterna magna accompanied by a cyst on brain MRI. Simultaneous WESs were performed using blood samples from the index patient, parents, maternal uncle, and maternal grandmother (Genetic testing was done as part of the screening and diagnosis of Marfan syndrome within the family members). A *de novo* heterozygous frameshift mutation (c.601delG/p.Ala201GlnfsTer148) in *SLC35A2* gene was found in the index patient only. P4 was also diagnosed to have Marfan syndrome due to carrying a known disease causing mutation in *FBN1* gene (NM_000138, heterozygous c.7240C > T/p.Arg2414Ter, HGMD CM020137), having typical features (congenital heart disease with VSD with mitral valve regurgitation, and arachnodactyly), and positive family history (mother and maternal grandmother) (Loeys et al., 2010). Normal test results at the age of 10 months include body weight (8 kg, 32nd centile), head circumference (42 cm, 5st centile), chest radiography, abdominal ultrasound, full blood count, serum biochemistry (including liver function test, serum creatinine, electrolytes, glucose, and transferrin levels), blood coagulation profiling, blood amino acid with acyl carnitine profiling, and urine organic acid analysis, and chromosome karyotyping (46XX).

**FIGURE 2:**
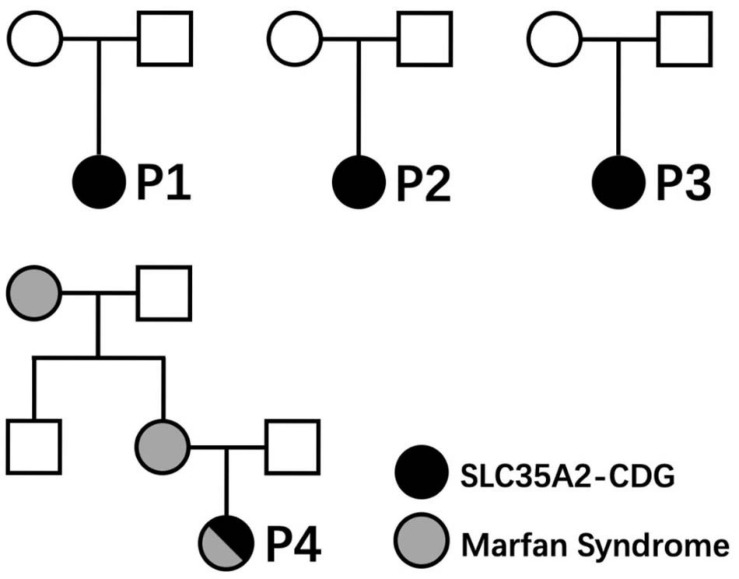
Pedigrees of patients reported in this article.

Clinical characteristics of patients from our cohort and previously reported cases are summarized in Table 1. None of those SLC35A2 variants are present in gnomAD including Chinese and East-Asian population. None of these variants had been reported to be associated with SLC35A2-CDG in English literature. All four variants were predicted to be damaging by various *in silico* pathogenicity prediction tools, and predicted to affect gene splicing by at least one splice-site prediction tool. On the other hand, splice-site, large deletion, and frameshift variants may also affect protein function by causing non-sense mediated mRNA decay or by skipping of exons during transcription. *SLC35A2* gene variants, carrier frequencies, and results of *in silico* prediction tools were provided in Table 2. Sanger sequencing or chromosome microarray testing results, transferrin iso-electric focusing results, location of variants on the gene, and predicted effect on protein domains were provided in Figure 1. We also provided pedigrees of all patients in Figure 2.

**TABLE 1:** Clinical characteristics of patients from our cohort and previous reports.

**Clinical phenotypes**	**Features in our report (underline, novel features)**				**Previously reported features in English literature**
**Patient ID**	**P1**	**P2**	**P3**	**P4**	
Gender, age	Female, 8.5 months	Female, 22 months	Female, 13 months	Female, 10 months	Female/Male 41/4 (Ng et al., 2019; Vals et al., 2019)
Growth percentiles	Weight 8th, height < 1st, Short stature	Weight 50th, head circumference 52nd	Weight 54th, height 37th	Weight 32nd, head circumference 5th	Short stature 67% (10/15) (The Undiagnosed Disease Network, 2016; Vals et al., 2019)
Developmental delay	Yes	Yes	None	Yes	100% (62/62) (Ng et al., 2019; Vals et al., 2019)
Hypotonia	−	No (hypertonia)	−	Yes	90% (54/60) (Ng et al., 2019; Vals et al., 2019)
Intellectual Disability	Yes	Yes	None	Yes	97% (29/30) (Ng et al., 2019; Vals et al., 2019)
Facial Dysmorphism	−	Smaller left ear with extra folds on both earlobes.	−	−	85% (53/62) (Ng et al., 2019; Vals et al., 2019)
Epilepsy	Yes	Yes	Yes	Yes	83% (52/63) (Ng et al., 2019; Vals et al., 2019)
Skeletal abnormalities	Short stature	−	−	Arachnodactyly due to Marfan syndrome	83% (43/52) Shortened limbs, short stature, contractures, scoliosis, clubfoot, pes adductus, and craniosynostosis (Ng et al., 2019; Vals et al., 2019)
Abnormal brain MRI	Yes (delayed myelination, enlargement of lateral ventricles and frontal subarachnoid spaces)	No	No	Yes (mega cisterna magna, and a brain cyst)	Cerebral atrophy (50%, 13/26), cerebellar atrophy (46%, 26/56), thin corpus callosum (39%, 23/56), and delayed/hypo-myelination (59%, 16/27) (Ng et al., 2019; Vals et al., 2019)
Gastrointestinal	−	−	−	−	Feeding problems (75%, 24/32), Gastric-tube feeding (69%, 22/32) (Dörre et al., 2015; Westenfield et al., 2018; Ng et al., 2019)
Failure to Thrive	Short stature	−	−	None	77% (23/30) (Ng et al., 2019)
Feeding problems	−	−	−	−	73% (22/30) (Ng et al., 2019)
Ocular abnormalities	Does not follow objects	−	−	−	75% (43/57), Cortical visual impairment, strabismus, refractive errors (Ng et al., 2019; Vals et al., 2019)
Skin	No	−	−	−	60% (18/30) Inverted nipples, hypo/hyper-pigmentation of the skin (Ng et al., 2019)
Liver	Neonatal hyperbilirubinemia	Neonatal hyperbilirubinemia	Neonatal hyperbilirubinemia	−	80% (24/30) Mildly elevated transaminases (ALT, AST, GGT) and AFP elevation, 40% (12/30) Neonatal hyperbilirubinemia (Ng et al., 2019)
Abnormal CDT	−	Yes	−	−	35% (11/30) Truncated N-glycans lacking galactose and terminal sialic acid (Ng et al., 2019; Vals et al., 2019)
Immunological	−	−	−	−	33% (10/30) (Ng et al., 2019)
Respiratory	−	−	−	−	33% (10/30) (Ng et al., 2019)
Heart	−	−	−	VSD and mitral valve regurgitation	27% (8/30) (Ng et al., 2019)
Hearing Loss	Does not follow sounds	−	−	−	23% (7/30), sensorineural hearing loss, impaired hearing (Ng et al., 2019)
Pregnancy complication	Conceptional oligohydramnios	Conceptional hypothyroidism	−	None	20% (fetal skeletal dysplasias, pericardial effusions, decreased fetal movements, breech presentation) (Ng et al., 2019)
Genital/Endocrine	None	−	−	−	17% (5/30), Premature signs of puberty, hypothyroidism, slight elevation of thyroid stimulating hormone and free T4) (Ng et al., 2019)
IUGR	Yes	None	−	None	13% (4/30) (Ng et al., 2019)
Kidney	Uric acidemia	−	−	None	3% (1/30) (Ng et al., 2019)
Other	Anemia, and hypertriglyceridemia		−	Marfan syndrome	tapering fingers (14%, 2/14), broad great toes or overlapped toes (20%, 3/15) (Dörre et al., 2015; Kimizu et al., 2017; Westenfield et al., 2018; Yates et al., 2018)

*−, not available or not tested; AFP, alphafetoprotein; CDT, carbohydrate-deficient transferrin test; IUGR, intrauterine growth restriction; VSD, ventrical septal defect; Underline, novel features.*

**TABLE 2:** SLC35A2 gene mutations, carrier frequency, and *in-silico* pathogenicity prediction results.

**Patients**			**P1**	**P2**	**P3**	**P4**
Physical location (GRCh37/hg19)			chrX:48763668C > T	chrX:48767090-48769235del	chrX:48762405 _48762405delG	chrX:48762585 _48762585delC
Nucleotide change (NM_001042498.2)			c.426+1G > A	Xp11.23del2.14kb (c.-322_c.274+1del, deletion of exon1 and exon2)	c.781delC	c.601delG
Amino acid change			NA	NA	p.Arg261ValfsTer88	p.Ala201GlnfsTer148
Parental origin			*De novo*	*De novo*	*De novo*	*De novo*
Frequency in control population		1000G	0	0	0	0
		ExAC	0	0	0	0
		gnomAD	0	0	0	0
Pathogenicity (ACMG criteria; Richards et al., 2015)			Class 5 (PS2+PM2+PM4 +PP3+PP4)	Class 5 (PS2+PS3+PM2 +PM4+PP3+PP4)	Class 5 (PS2+PM2+PM4 +PP3+PP4)	Class 5 (PS2+PM2+PM4 +PP3+PP4)
*In silico* prediction of pathogenicity (Score)	Mutation Taster		Disease causing (1)	NA	Disease causing (1)	Disease causing (1)
	M-CAP		Possibly pathogenic (0.838)	NA	NA	NA
	CADD		Pathogenic (34)	NA	NA	NA
	SIFT Indel		NA	Non-sense Mediated Decay	Damaging	Damaging
Effect of variants on gene splicing	BDGP/NNSplice		Donor site affected	Donor and acceptor sites affected	Donor and acceptor sites affected	None
	ESE Finder 3.0		Broken WT Donor Site	Loss of BranchSite	ESE site affected	None
	HSF3.1		New Donor Site, New ESS Site	ESS Sites broken, New ESE Site	None	None
	Mutation Taster		Donor lost, Acc increased	NA	Acc increased	Donor increased/gained
	ASSP		None	None	None	None
Predicted effect			Splice variant	Large deletion	Frameshift,	Frameshift

*ASSP, Alternative Splice Site Predictor; BDGP/NNSplice, Berkeley Drosophila Genome Project/Splice Site Prediction by Neural Network; ESE, exonic splicing enhancer; ESS, exonic splicing silencer; HSF, Human Splicing Finder; NA, not available or not applicable; Pathogenicity thresholds: MutationTaster (probability), M-CAP (> 0.025), CADD (> 20).*

## Discussion

We presented four new patients with typical features of SLC35A2-CDG carrying *de novo* and deleterious mutations in *SLC35A2* gene.

Previously reported facial dysmorphisms included brachycephaly, coarse face, hypertelorism, high-set/broad/thick eyebrows, large blue irises, long narrow palpebral fissures, eversion of the lower lids, long eyelashes, epicanthal folds, low-set/posteriorly-rotated ears, mid-face hypoplasia, full cheek, depressed/broad nasal bridge, short nose, upturned nares, hypoplastic alae nasi, long, or short philtrum, thick lips, prominent cupid’s bow, protruding tongue, fused tooth, enamel hypoplasia, high-arched palate, maxillary prognathism, and retrognathia (Kodera et al., 2013; Dörre et al., 2015; Westenfield et al., 2018). One patient in our cohort (P2) had smaller left ear with extra folds on both earlobes, a potentially new dysmorphism associated with SLC35A2-CDG.

Slight elevation of liver enzyme, hypothyroidism (Vals et al., 2019), and some fetal abnormalities (fetal skeletal dysplasias, pericardial effusion, decreased fetal movements, and breech presentation) (Ng et al., 2019) had been reported before. However, one patient (P1) in our cohort had anemia and hypertriglyceridemia, while another patient (P2) had hypertonia. All of which have never been reported in previous reports and could be novel phenotypes of SLC35A2-CDG. Previously reported pregnancy complications included fetal skeletal dysplasias, pericardial effusions, decreased fetal movements, and breech presentation. New findings on pregnancy complications in our cohort include conceptional oligohydramnios (P1) and conceptional hypothyroidism (P2). For the first time, we reported an SLC35A2-CDG patient with concomitant Marfan syndrome (P4).

Previously reported patients had various types of brain MRI abnormalities, including white matter abnormalities, cerebral/cerebellar/brainstem/pons atrophy, thin/short corpus callosum, delayed/hypo-myelination, frontal cortical dysplasia, diffusion abnormalities in mesencephalon/dentate, nucleus/cerebellar peduncule, Dandy-Walker malformation (congenital hydrocephalus affecting the cerebellum), arachnoidal pouch, periventricular frontal nodules, and enlarged lateral ventricle. P1 had delayed myelination, enlargement of lateral ventricles and frontal subarachnoid spaces the other hand, P4 had mega cisterna magna, and a brain cyst probably due to the coexistence of Marfan syndrome.

According to 2015 American College of Medical Genetics and Genomics (ACMG) guideline (Richards et al., 2015), c.426+1G > A (*de novo* PS2 + absent from controls PM2 + change in protein length PM4 + *in silico* evidence PP3 + phenotype match PP4), c.-322_c.274+1del (*de novo* PS2 + functional test PS3 + absent from controls PM2 + change in protein length PM4 + *in silico* evidence PP3 + phenotype match PP4), c.781delC/p.Arg261ValfsTer88 (*de novo* PS2 + absent from controls PM2 + change in protein length PM4 + *in silico* evidence PP3 + phenotype match PP4), and c.601delG/p.Ala201GlnfsTer148 (*de novo* PS2 + absent from controls PM2 + change in protein length PM4 + *in silico* evidence PP3 + phenotype match PP4) are all classified as Class 5 pathogenic variants. Two glycine (Gly202 and Gly214) and lysine residues (Lys78 and Lys297) are critical for transporter activity of SLC35A2 protein (Li and Mukhopadhyay, 2019). Transport activities in our patients could be completely absent due to Gly202/Gly214/Lys297 loss in P1 and P4 (143-388 amino acid residues could be absent due to loss of exon 4 caused by c.426+1G > A in P1, and amino acid sequence is completely changed beginning with Ala201 in P4 due to p.Ala201GlnfsTer148), Lys78 loss in P2 (1-91 amino acid residues could be absent due to exon 1 and exon 2 deletion caused by c.-322_c.274+1del), Lys297 loss in P3 (p.Arg261ValfsTer88). Canonical splice-site mutations (c.-322_c.274+1del), large deletions (c.781delC/p.Arg261ValfsTer88), and frameshift mutations (c.781delC/p.Arg261ValfsTer88 and c.601delG/p.Ala201GlnfsTer148) may also cause absence of the protein product through non-sense-mediated mRNA decay (NMD) (Hu and Ng, 2012). Previously, only two splice site mutations have been reported (c.274+2T > C and c.274+1T > C) to be associated with SLC35A2-CDG (Miyamoto et al., 2019; Ng et al., 2019). The c.426+1G > A variant in P1 is a canonical exon 3 splicing donor site mutation that usually causes skipping of Exon 4 during transcription and translation (Maszczak-Seneczko et al., 2011; Shibata et al., 2016; Anna and Monika, 2018) leading to loss of transmembrane domains (TMD) 5–10. All variants found in our patient caused amino acid changes in strictly conserved positions within TMDs in SLC35A2 protein by frameshifting or deletion, predicted to affect at least one feature of gene splicing, and predicted to be disease causing by multiple pathogenicity prediction tools (Table 2). In Table 3, we also listed all *SLC35A2* gene mutations reported so far.

**TABLE 3:** Reported *SLC35A2* gene mutations (NM_001042498.2) in order of cDNA change.

**cDNA change (NM_001042498.2)**	**Amino acid change**	**References**
c.-322_c.274+1del	?	Current report
c.1A > G	Met1?	Ng et al., 2019
c.3G > A	Met1?	Ng et al., 2013
c.15_91+48delinsA	Gly8Serfs*9	Ng et al., 2013
c.124del	Val42Cysfs*53	Vals et al., 2019
c.164G > C	Arg55Pro	Ng et al., 2019; Vals et al., 2019
c.168C > A	Tyr56Ter	Ng et al., 2019
c.193_204del	Phe65_Thr68del	Ng et al., 2019
c.195C > A	Phe65Leu	Yates et al., 2018
c.211G > A	Val71Met	Ng et al., 2019
c.245G > T	Cys82Phe	The Undiagnosed Disease Network, 2016; Ng et al., 2019
c.262G > C	Ala88Pro	Vals et al., 2019
c.274+1G > A	?	Miyamoto et al., 2019
c.274+2T > C	?	Ng et al., 2019
c.302T > C	Leu101Pro	Ng et al., 2019
c.327T > G	Tyr106Ter	Yates et al., 2018
c.346G > C	p.Ala116Pro	Ng et al., 2019
c.348del	Val117Cysfs*27	Ng et al., 2019
c.353C > G	Pro118Arg	Ng et al., 2019
c.389A > G	Tyr130Cys	Ng et al., 2019; Vals et al., 2019
c.426+1G > A	?	Current report
c.433_434del	Tyr145Profs*76	Kodera et al., 2013
c.466_468delTCC	Ser156del	Vals et al., 2019
c.497_501dup	Gln168Glyfs*183	Ng et al., 2019
c.502C > T	Gln168Ter	EuroEPINOMICS-Res Consortium, Epilepsy Phenome/Genome Project, and Epi4K Consortium., 2014; Ng et al., 2019
c.515T > C	Leu172Pro	Yates et al., 2018
c.523C > T	Leu175Phe	Ng et al., 2019
c.523_525del	Leu175del	Ng et al., 2019
c.547C > T	Gln183Ter	Ng et al., 2019
c.562G > A	Gly188Ser	Ng et al., 2019
c.569dup	Gly191Argfs*31	Ng et al., 2019
c.601delG	Ala201Glnfs*148	Current report
c.617del	Val206Alafs*143	Ng et al., 2019
c.638C > T	Ser213Phe	Kodera et al., 2013
c.670C > T	Leu224Phe	Vals et al., 2019
c.683C > A	Ser228Ter	EuroEPINOMICS-Res Consortium, Epilepsy Phenome/Genome Project, and Epi4K Consortium., 2014
c.698T > C	Leu233Pro	Ng et al., 2019; Vals et al., 2019
c.747_757dup	Ala253Glyfs*100	Ng et al., 2019
c.753delG	Trp251Cysfs*98	Vals et al., 2019
c.772G > A	Val258Met	Lopes et al., 2016; Vals et al., 2019
c.781delC	Arg261Valfs*88	Current report
c.795del	Phe265Leufs*84	Ng et al., 2019
c.797G > T	Gly266Val	Dörre et al., 2015
c.800A > G	Tyr267Cys	Bosch et al., 2016; Vals et al., 2019
c.816G > A	Trp272Ter	Ng et al., 2019
c.818G > A	Gly273Asp	Ng et al., 2019; Vals et al., 2019
c.831C > G	Asn277Lys	Evers et al., 2017
c.841G > A	Gly281Ser	Vals et al., 2019
c.841G > C	Gly281Arg	Vals et al., 2019
c.856del	Ala286Leufs*63	Ng et al., 2019
c.889A > G	Lys297Glu	Yates et al., 2018
c.908T > C	Leu303Pro	Ng et al., 2019
c.923C > T	Ser308Phe	Yates et al., 2018; Vals et al., 2019
c.935C > A	Ser312Tyr	Ng et al., 2019
c.944T > C	Leu315Pro	Ng et al., 2019
c.950delG	Gly317Alafs*32	Kimizu et al., 2017
c.972del	Phe324Leufs*25	Kodera et al., 2013
c.991G > A	Val331Ile	Ng et al., 2013, 2019; Westenfield et al., 2018; Vals et al., 2019

All patients were females in our cohort, and all carried deleterious mutations. Ng et al. (2013) calculated significantly more female patients with SLC35A2-CDG carried deleterious mutations (41%) than male patients (25%), indicating deleterious mutations might be less tolerated in male patients and retention of SLC35A2 protein functionality (either in the form of missense variant or mosaicism) might be needed for survival in male patients.

Transferrin isoelectric focusing (IEF) in some patients with SLC35A2-CDG showed type II patterns with increased levels of hypoglycosylated transferrins along with decreased level of tetrasialated transferrin (Ng et al., 2013; Dörre et al., 2015). However, transferrin IEF screening in some patients showed normal patterns during earlier or later years of disease course (9 out of 15) (Vals et al., 2019). P2 in our cohort had abnormal type II pattern with increased levels of asialated, monosialated, disialated, and trisialated transferrins. Mass spectrometry (MS) of N-glycans showed loss of galactose in one study (Vals et al., 2019), and partial loss of galactose and sialic acid of the N−linked glycans of serum transferrin was observed in other studies (Ng et al., 2013; Miyamoto et al., 2019).

Assays using Streptolysin-O-permeabilized fibroblasts from patients (Ng et al., 2013), glycoside−based UDP−galactose transport assays with primary fibroblast lines from patients (Ng et al., 2019), or a novel assay using the binding of bacterial Shiga toxins (Li and Mukhopadhyay, 2019) can be used to evaluate SLC35A2 enzyme activity. Deleterious mutations in our cohort have high likelihood of leading to complete loss of enzyme activity. Effects of variants found in our patients on enzyme activity need to be confirmed by similar assays.

There is no disease-specific treatment for SLC35A2-CDG that proven to be effective, but oral galactose supplementation may improve residual enzyme activity by providing extra substrate. Dörre et al. (2015) reported reduced seizure activity and improved transferrin IEF pattern after 100 days of oral galactose therapy (1 g/kg^∗^day, divided into five doses) in a 6-months-old infant. Same efficacy may be achieved when the galactose was administered as a single morning dosage without having additional side effect (Witters et al., 2020). P2 and P3 achieved seizure control with minimal EEG abnormality after gradual increase of oral D-galactose up to 1.5 g/kg^∗^day (starting with 0.5 g/kg^∗^day and adding 0.5 g/kg^∗^day in 6 weeks). P4 had improved motor development, reduced seizure activity, and improved EEG patterns after receiving 1 g/kg^∗^day (divided to eight doses in a day) of oral galactose supplement.

Due to retrospective nature of this study, some limitations could not be avoided. Limitations in our study include lack of functional studies to confirm effects of mutations on protein function, N-glycan analysis for the confirmation of glycosylation defect, and transferrin IEF for P1, P3, and P4 for the screening of glycosylation defect. Nevertheless, we believe that the available information should be sufficient for the diagnosis of SLC35A2-CDG.

In summary, we expanded genotypic and phenotypic spectrum of SLC35A2-CD by reporting first four patients with novel *SLC35A2* gene variants and novel phenotypes from mainland China.

## Data Availability Statement

The original contributions presented in the study are included in the article/supplementary material, further inquiries can be directed to the corresponding author/s.

## Ethics Statement

The studies involving human participants were reviewed and approved by the Ethics Committee of the Children’s Hospital of Fudan University. Written informed consent to participate in this study was provided by the participants’ legal guardian/next of kin. Written informed consent was obtained from the individual(s), and minor(s)’ legal guardian/next of kin, for the publication of any potentially identifiable images or data included in this article.

## Author Contributions

J-SW and KA designed the study, approved the final submission, and clinically managed patients. KA collected clinical and genetic data, performed *in-silico* prediction, conducted literature search, summarized relevant information, and wrote the manuscript. Both authors contributed to the article and approved the submitted version.

## Conflict of Interest

The authors declare that the research was conducted in the absence of any commercial or financial relationships that could be construed as a potential conflict of interest.

## Footnotes

1 https://cnki.net

2 www.wanfangdata.com.cn

3 www.hgmd.cf.ac.uk/

4 www.findrare.cn

5 www.berrygenomics.com

6 www.kangso.net

7 www.ncbi.nlm.nih.gov/snp

8 www.1000genomes.org/

9 http://exac.broadinstitute.org

10 http://gnomad.broadinstitute.org

11 https://mutalyzer.nl/position-converter

12 www.mutationtaster.org

13 https://cadd.gs.washington.edu

14 https://sift.bii.a-star.edu.sg/www/SIFT_indels2.html

15 http://bejerano.stanford.edu/MCAP/

16 www.fruitfly.org/seq_tools/splice.html

17 http://krainer01.cshl.edu/cgi-bin/tools/ESE3/esefinder.cgi

18 www.umd.be/HSF

19 http://wangcomputing.com/assp/index.html

20 https://vita-world24.de/ihre-ziele/jung-vital-schoen/117/d-galactose-500-g

## Abbreviations

ACTH: adrenocorticotropic hormone
AEDs: anti-epileptic drugs
AFP: alphafetoprotein
ALT: alanine aminotransferase
AST: aspartate aminotransferase
BAEP: brainstem auditory evoked potential
CDG: congenital disorders of glycosylation
CDT: carbohydrate-deficient transferrin test
CMV: cytomegalovirus
CNKI: China Knowledge Resource Integrated Database
CT: computed tomography
EEG: electroencephalogram
GGT: gamma-glutamyl transpeptidase
IUGR: intrauterine growth restriction
MRI: magnetic resonance imaging
NMD: non-sense-mediated mRNA decay
TSH: thyroid stimulating hormone
VEP: visual evoked potential.
